# Lean body mass as an independent determinant of dose‐limiting toxicity and neuropathy in patients with colon cancer treated with FOLFOX regimens

**DOI:** 10.1002/cam4.621

**Published:** 2016-01-27

**Authors:** Raafi Ali, Vickie E. Baracos, Michael B. Sawyer, Laurent Bianchi, Sarah Roberts, Eric Assenat, Caroline Mollevi, Pierre Senesse

**Affiliations:** ^1^ Department of OncologyUniversity of AlbertaEdmontonCanada; ^2^ Department of Clinical Nutrition and GastroenterologyInstitute of Cancer MontpellierMontpellierFrance; ^3^ Department of GastroenterologyUniversity Hospital Montpellier FranceMontpellierFrance; ^4^ Biometrics Unit – CTD INCaInstitut régional du Cancer de Montpellier (ICM)Montpellier France; ^5^ SIRIC Montpellier CancerInstitut régional du Cancer de Montpellier (ICM)MontpellierFrance

**Keywords:** Body composition, chemotherapy toxicity, colon cancer, irinotecan, lean body mass, neuropathy, oxaliplatin

## Abstract

Evidence suggests that lean body mass (LBM) may be useful to normalize chemotherapy doses. Data from one prospective and one retrospective study were used to determine if the highest doses of oxaliplatin/kg LBM within FOLFOX regimens would be associated with dose‐limiting toxicity (DLT) in colon cancer patients. Toxicity over four cycles was graded according to NCI Common Toxicity Criteria V2 or V3 (Common Terminology Criteria for Adverse Events, National Cancer Institute, Bethesda, MD). Muscle tissue was measured by computerized tomography (CT) and used to evaluate the LBM compartment of the whole body. In prospective randomized clinical trials conducted in France (*n *=* *58), for patients given FOLFOX‐based regimens according to body surface area, values of oxaliplatin/kg LBM were highly variable, ranging from 2.55 to 6.6 mg/kg LBM. A cut point of 3.09 mg oxaliplatin/kg LBM for developing toxicity was determined by Receiver Operating Characteristic (ROC) analysis, below this value 0/17 (0.0%) of patients experienced DLT; in contrast above this value 18/41 (44.0%) of patients were dose reduced or had treatment terminated owing to toxicity (≥Grade 3 or neuropathy ≥Grade 2); for 9/41 the DLT was sensory neuropathy. These findings were validated in an independent cohort of colon cancer patients (*n *=* *80) receiving FOLFOX regimens as part of standard care, in Canada. Low LBM is a significant predictor of toxicity and neuropathy in patients administered FOLFOX‐based regimens using conventional body surface area (BSA) dosing.

## Introduction

For many cancer drugs dose is scaled to body surface area (BSA). This convention began with observations that basal metabolic rates scaled between species according to weight. BSA was used to estimate an appropriate starting dose for an anticancer drug for phase I studies based on preclinical animal studies 1. BSA dosing became established in medical oncology without strong evidence that pharmacokinetic interpatient variation correlated with BSA and it has been argued that dose adjustment of chemotherapy by BSA does not necessarily reduce toxicity 1, 2, 3, 4, 5.

Interpatient variation in toxicities can arise from differences in target protein(s) expression, drug metabolism, and excretion. Disparate metabolism and excretion of anticancer drugs in turn can be due to environmental, physiologic, and genetic factors. Heterogeneous body composition of cancer patients (i.e., relative amounts of lean and adipose tissue) has also been suggested to contribute to interpatient variation in toxicities 2, 3, 6, 7, 8. The rationale is that the overall weight is comprised of two major compartments (fat and lean), which may be the major sites of distribution of lipophilic‐ and nonlipophilic drugs, respectively. The lean compartment is comprised of metabolic tissues such as the liver and kidney 2, 9, intra and extracellular water, skeletal muscle, and bone. Lean body mass (LBM) has been suggested several times 2, 3, 6, 7, 8 to be of particular relevance of anticancer agents that distribute in and are metabolized within the lean compartment and some of these authors raised the suggestion of potentially normalizing chemotherapy doses to LBM.

**Figure 1:**
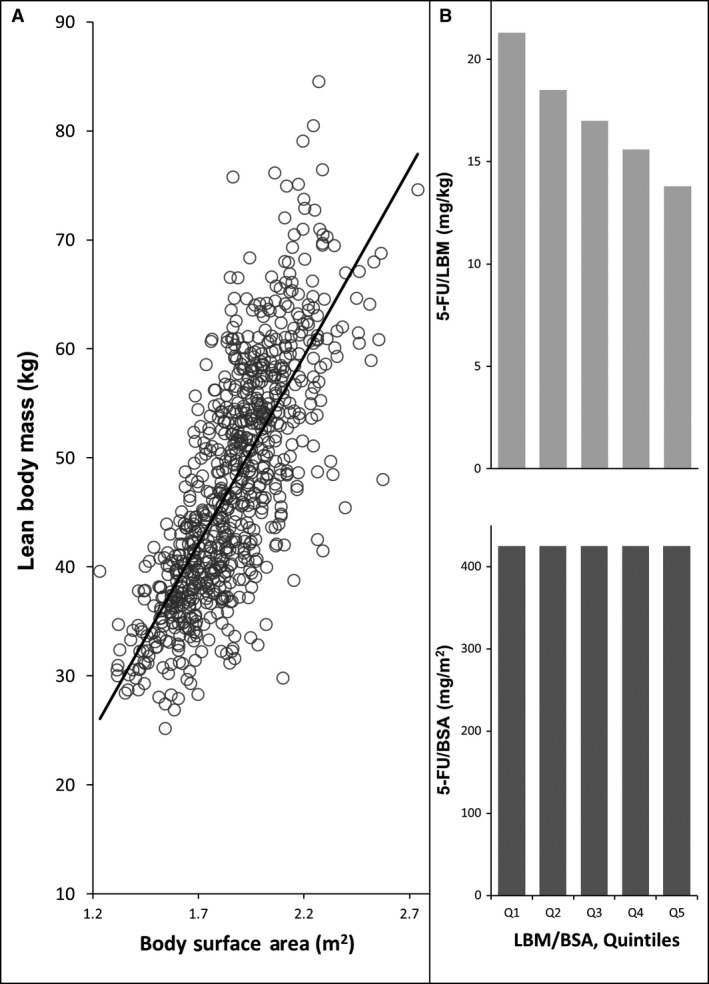
Relationship between computed tomography defined lean body mass (LBM) and body surface area (BSA) in 776 colorectal cancer patients referred to a medical oncology service in northern Alberta, Canada. (A) Shows a weak relationship between BSA and LBM, *R* ^*2 *^= 0.5341. (B) Potential effect of body composition across quintiles of the ratio of LBM/BSA. Upper panel shows the potential variation in 5‐Fluorouracil (5FU)/kg LBM that would result in this population as a consequence of the variation in body composition, if all patients were to be administered 425 mg/m^2^ of 5FU (lower panel).

There are very considerable variations in LBM in patients of identical BSA. This has been documented by the application of computed‐tomography (CT) to specifically and precisely quantify lean and fat mass in cancer patients. LBM was shown to be weakly correlated with BSA (*r* ^*2*^ = 0.37) in obese patients with solid tumors 10. This rather poor correlation results from the fact that some obese patients are very muscular, some are affected by severe depletion of the LBM (i.e., sarcopenic obesity) and thus have a very low LBM relative to their BSA. This variation can be illustrated with our data from a large (*n *=* *1473) population cohort 11 of patients with cancers of the colon and rectum referred to a regional medical oncology service (Fig. 1). BSA shows a weak relationship with CT‐defined LBM (*r* ^*2*^ = 0.54) (Fig. 1A). The potential impact of this variation is underscored by calculating the drug dose/kg LBM that may result if these patients were to be given BSA‐based doses of 5‐fluorouracil (5FU) (for purposes of example: 425 mg/m²). People in the top quintile of LBM/BSA would theoretically receive 13.8 mg of 5FU/kg LBM, whereas those in the bottom quintile would receive of 21.3 mg/kg LBM (a difference of 53%) (Fig 1B, upper panel) even though all patients would receive 425 mg/m^2^ of BSA (Fig 1B, lower panel). Owing to gender‐specific characteristic (i.e., men have more muscle), the lowest quintile includes a preponderance of females and the highest quintile mainly males.

Based on the foregoing, it would be predicted that individuals with low LBM relative to their BSA, might be at risk for excess toxicity owing to a concentration of the drug in the LBM. There is an emerging literature in which these predictions have been studied 12, 13, 14, 15, 16. For example, low LBM was a significant predictor of dose‐limiting toxicity (DLT) in colon cancer patients administered single agent 5FU using conventional BSA‐based dosing and DLT was concentrated in patients who received >20 mg 5FU/kg LBM, the majority of whom were female 12. Conversely, the incidence of DLT was very low in patients from the same population falling below that cut point. Likewise, as multiple agents with a given regimen may all be administered per unit BSA, patients whose LBM is low relative to their BSA, may receive effectively a higher dose of all constituents of the regimen, placing them at risk for increased severe toxicity. For example, nonsmall cell lung cancer patients treated with a BSA‐based regimen received gemcitabine varying from 23.2 to 53.1 mg/kg LBM, and vinorelbine from 1.5 to 3.3 mg/kg LBM, and higher doses of these agents per kg LBM significantly associated with grade 3–4 hematological toxicity 13. In addition to the examples given above, excess toxicity was seen in patients with a low LBM relative to their BSA, across a variety of cancer therapies and this was summarized in a recent review 14. This excess toxicity was seen with targeted agents (sunitinib, sorafenib, vandetanib 14, afatinib 15) as well as neoadjuvant combinations 16 and single agents such as capecitabine 17. While these studies had consistent conclusions, they had small samples sizes, were retrospective in design, conducted in single centers and did not include any validation cohort.

Our aim was to further assess the potential for excess toxicity associated with low LBM in patients treated with an established chemotherapy regimen used in colorectal cancer, FOLFOX, with particular attention to one of its specific toxicities, sensory neuropathy. We evaluated this in two independent populations of patients.

## Materials and Methods

### Patients and study design

Patients in all studies had a histologically proven diagnosis of cancer of the ascending, transverse or descending colon, rectum, or recto‐sigmoid junction. All studies were approved by relevant Research Ethics Boards in France (Comité de protection des personnes sud méditerranée IV) and Canada (Health Research Ethics Board of Alberta‐Cancer). BSA was calculated using the Mosteller formula [BSA (m^2^) = ([Height (cm) × Weight (kg)]/3600)^1/2^]. All patients received regimens including folinic acid (200 mg/m²), 5FU bolus (400 mg/m²), infusional 5FU 46 h (2400 mg/m²), biweekly for up to 12 cycles combined with oxaliplatin, irinotecan and/or cetuximab.

#### Study population I – France

Included patients were participants in two prospectively conducted multicentre Phase II clinical trials in France, providing a defined population of patients with metastatic disease and good performance status (0 or 1). The METHEP study (NCT00208260, *Intensified Chemotherapy in Colorectal Cancer After Resection of Liver Metastases*)^18^ compared standard FOLFOX‐4 and FOLFIRI, with intensified treatments (FOLFOX‐7, high‐dose FOLFIRI, FOLFIRINOX). The ERBIRINOX study (NCT00556413, *Cetuximab and Combination Chemotherapy as First‐Line Therapy in Treating Patients With Metastatic Colorectal Cancer*)^19^ evaluated the efficacy and toxicity of FOLFIRINOX with and without cetuximab. Treatment toxicity was prospectively collected at each cycle according to NCI‐CTCAE (Common Terminology Criteria for Adverse Events, National Cancer Institute, Bethesda, MD) v2.0 for the patients in METHEP, and v3.0 for those in ERBIRINOX. Sensory neuropathy was evaluated using the modified Levi scale. For the present analyses, toxicity data recorded per protocol was used. Toxicity was considered dose limiting if it was ≥Grade 3 (or ≥Grade 2 for neuropathy) and was the basis for a decision to reduce or terminate therapy. Toxicity during the first four cycles was considered in the main analysis.

All patients received regimens based on folinic acid (200 mg/m²), 5FU bolus (400 mg/m²), infusional 5FU 46 h (2400 mg/m²), biweekly for up to 12 cycles, combined with oxaliplatin (85 mg/m², or 130 mg/m²). ERBIRINOX also included irinotecan : 180 mg/m² on top of oxaliplatin 85 mg/m², with or without cetuximab 400 mg/m².

#### Study population II – Canada

This group was a population cohort of consecutively referred patients receiving standard care for colorectal cancer at a Medical Oncology department at regional cancer center serving northern Alberta, Canada. This population was chosen to test whether findings from the clinical trials could be validated in an unselected population receiving standard of care. All patients received regimens based on folinic acid (200 mg/m²), 5FU bolus (400 mg/m²), infusional 5FU 46 h (2400 mg/m²), biweekly for up to 12 cycles combined with oxaliplatin. Patients with metastatic disease received standard FOLFOX (100 mg/m^2^ of oxaliplatin). Patients with early stage disease received FOLFOX (85) oxaliplatin at 85 mg/m^2^.

In this *standard care* sample, chemotherapy administration records of the hospital pharmacy were reviewed for physician‐ordered dose reductions and termination of current therapy. DLT were recorded in instances where these changes in treatment plan were ordered by the medical oncologist for the specific reason of treatment‐related toxicity; the type of toxicity was recorded. Patient toxicity assessments were obtained through physician notes, after each cycle of chemotherapy and this was reviewed by a research nurse. Progressive disease as the cause of termination of therapy was not recorded as DLT. Only DLT experienced within the first four cycles were evaluated in the main analysis, as was done for the French cohort.

### Body composition measurements

#### Anthropometric measurements

Weight and height were recorded during visits according to standard methods. Weight was measured with a medical balance beam scale and height was measured with a stadiometer. Body mass index (BMI) was calculated (weight (kg)/height (m^2^)).

#### Image analysis

Computerized tomography scans completed with a spiral CT scanner for initial cancer staging and routine diagnostic purposes were used to quantify skeletal muscle area. Cross‐sectional imaging using CT or magnetic resonance imaging is suggested as the preferred method for analyzing muscle mass in patients with cancer 20, 21. CT scans completed within 30 days of the beginning of treatment were deemed to accurately represent baseline body composition. Two adjacent axial images within the same series, at the 3rd lumbar vertebra, were selected for analysis of total muscle cross‐sectional area (cm^2^) and averaged for each patient 22, 23, 24, 25. CT image parameters included: contrast‐enhanced, 5‐mm slice thickness, 120 kVp, and ~290 mA. Observers were blinded to patients treatment and toxicity status. Muscles were quantified within a Hounsfield unit(HU) range of −29 to +150 HU using Slice‐O‐Matic software (v.4.3;Tomovision, Montreal, Quebec, Canada). Muscle area was normalized for height in meters squared (m^2^) and reported as lumbar SMI(cm^2^/m^2^) 23. The formula used to calculate whole‐body LBM (kg) = [((L3 Muscle measured by CT (cm^2^) × 0.3) + 6.06]. The coefficient of correlation of this regression, *r* = 0.94) 23.

### Statistics and analysis plan

To represent the dose—intensity of the regimen overall and because oxaliplatin—associated neuropathy is a key toxicity of this regimen, the absolute dose of oxaliplatin (mg) given according to treatment plan at cycle one was normalized to lean body mass, for each patient. This rendered a continuous variable (estimated oxaliplatin dose/kg LBM) which ranged from 2.55 to 6.60 mg/kg LBM in the French cohort and from 2.68 to 5.00 mg/kg LBM in the Canadian cohort. Data in this form were tested for the presence of a cut point (i.e., a threshold value of drug/kg LBM) defining increased risk of DLT using ROC analysis 26.

Descriptive data were expressed as mean ± SD and comparisons between two means were made by Student's *t*‐test for unpaired data. Significance of the association between two categorical variables was assessed by Fisher's exact test. All *P*‐values were two‐sided and levels of significance were *P *<* *0.05. Statistical analysis was done using SPSS (SPSS for Windows, version 14.0, SPSS, Chicago, IL). STATA (StataCorp. 2013. Stata Statistical Software: Release 13. College Station, TX: StataCorp LP) data analysis and statistical software was used for cut‐point analysis.

## Results

### French cohort

Table 1 describes the patients' demographics, disease characteristics, chemotherapy regimens, body composition, and DLTs. For all regimens the administered dose was within ~5% of the target dose. Body weight and composition features showed variation between and within sex although women are generally less muscular and have lower LBM than men. Overall 31.0% of patients had dose reductions within the first four cycles or ended treatment owing to toxicity before fourth cycle. Overall, 51.9% of women and 12.9% of men experienced DLT within the first four cycles (*P *=* *0.0014, Fisher's Exact Test).

**Table 1:** Patients characteristics and rates of toxicity

Variables	France		Canada		Total
	Females	Males	Females	Males	
Number of patients	27	31	38	42	138
Patient characteristics^a^					
Age	59.6 ± 10.5	60.0 ± 6.2	60.8 ± 10.6	64.6 ± 11.8	61.5 ± 10.3
Weight (kg)	59.7 ± 10.6	77.3 ± 13.2	68.1 ± 14.2	81.5 ± 13.2	72.6 ± 15.3
Height (cm)	159 ± 6.7	174 ± 6.7	177 ± 6.5	161 ± 6.4	168 ± 10.0
Body mass index (kg/m^2^)	23.6 ± 4.1	25.5 ± 3.4	26.3 ± 5.3	26.1 ± 3.7	25.5 ± 4.3
<18.5 Underweight	2 (7.4%)	0 (0.0%)	4 (10.5%)	0 (0.0%)	6 (4.3%)
18.5–5.0 Normal	16 (59.3%)	16 (51.6%)	14 (36.8%)	17 (40.5%)	63 (45.7%)
25.0 – 30.0 Overweight	7 (25.9%)	13 (41.9%)	13 (34.2%)	18 (42.9%)	51 (37.0%)
>30.0 Obese	2 (7.4%)	2 (6.5%)	7 (18.4%)	7 (16.7%)	18 (13.0%)
Body surface area (m^2^) Sarcopenia	1.62 ± 0.16	1.92 ± 0.19	1.71 ± 0.17	1.99 ± 0.17	1.82 ± 0.22
Yes	16 (59.3%)	1 (3.2%)	22 (57.9%)	23 (54.8%)	62 (44.9%)
No	11 (40.7%)	30 (96.8%)	16 (42.1%)	19 (45.2%)	76 (55.1%)
Total muscle area (cm^2^)	102.9 ± 15.7	158.7 ± 20.5	110.4 ± 21.0	154.8 ± 24.6	133.3 ± 32.7
Total fat area (cm^2^)	195.0 ± 111	294.9 ± 147	301 ± 167	371 ± 164	297.8 ± 164
Muscularity (cm^2^/m^2^)	40.5 ± 5.2	52.6 ± 5.5	42.5 ± 6.7	49.5 ± 7.7	46.5 ± 8.0
Fatness (cm^2^/m^2^)	76.6 ± 44.2	97.4 ± 47.3	116.9 ± 66.2	118.9 ± 52.1	104.3 ± 56.2
Whole‐body lean mass (kg)	36.9 ± 4.7	53.7 ± 6.2	39.2 ± 6.3	52.5 ± 7.7	46.0 ± 9.8
Muscle attenuation (HU)	41.6 ± 10.7	41.8 ± 8.3	34.4 ± 8.9	31.6 ± 8.7	36.6 ± 10.0
Visceral adipose area (cm^2^)	52.9 ± 45.8	171.2 ± 109	96.4 ± 78.9	183.1 ± 110.5	131.1 ± 105.2
Cancer primary site					
Ascending colon	10 (37.0%)	6 (19.3%)	14 (36.8%)	9 (21.4%)	39 (28.3%)
Transverse colon	1 (3.7%)	3 (9.7%)	5 (13.2%)	4 (9.5%)	13 (9.4%)
Descending colon	9 (33.3%)	11 (35.5%)	10 (26.3%)	14 (33.3%)	44 (31.9%)
Rectum	4 (14.8%)	4 (12.9%)	2 (5.3%)	9 (21.4%)|	19 (13.8%)
Rectosigmoid junction	3 (11.1%)	7 (22.6%)	7 (18.4%)	6 (14.3%)	22 (15.9%)
Disease stage (IV) %	100%	100%	60.5%	45.2%	–
Functional status 0, 1, 2, 3 (%)	59.3%, 40.7%, 0.0%, 0.0%	74.2%, 25.8%, 0.0%0.0%	11.8%, 58.8%, 17.6%, 11.8%	34.8%, 60.9%, 4.3%, 0.0%	–
Treatment regimen					
FOLFOX	6 (22.2%)	6 (19.4%)	0 (0.0%)	0 (0.0%)	12 (8.7%)
FOLFIRINOX	9 (33.3%)	9 (29.0%)	0 (0.0%)	0 (0.0%)	18 (13.0%)
FOLFIRINOX+CETUXIMAB	12 (44.4%)	16 (51.6%)	0 (0.0%)	0 (0.0%)	28 (20.3%)
FOLFOX (100)	0 (0.0%)	0 (0.0%)	25 (65.8%)	34 (80.9%)	59 (42.8%)
Colon ADJ FOLFOX (85)	0 (0.0%)	0 (0.0%)	13 (34.2%)	8 (19.1%)	21 (15.2%)
Oxaliplatin (mg/kg LBM)	3.9 ± 0.8	3.3 ± 0.7	4.3 ± 0.6	3.6 ± 0.5	3.8 ± 0.72
5FU (mg/kg LBM)	119.3 ± 19.6	99.4 ± 10.3	124.6 ± 12.2	104.5 ± 14.3	111.8 ± 17.5
Dose‐limiting toxicity, *n* (%)					
Mucositis	2 (7.4%)	0 (0.0%)	1 (2.6%)	0 (0.0%)	3 (3.4%)
Diarrhea	2 (7.4%)	0 (0.0%)	4 (10.5%)	2 (4.8%)	8 (3.4%)
Neuropathy	7 (25.9%)	2 (6.5%)	3 (7.9%)	6 (14.3%)	18 (13.0%)
Neutropenia	2 (7.4%)	0 (0.0%)	5 (13.2)	3 (7.1%)	10 (2.2%)
Anemia	1 (3.7%)	1 (3.2%)	0 (0.0%)	0 (0.0%)	2 (1.4%)
Nausea/Vomiting	2 (7.4%)	2 (6.5%)	6 (15.8%)	2 (4.2%)	12 (8.7%)
Anorexia	2 (7.4%)	1 (3.2%)	2 (5.3%)	2 (4.2%)	7 (5.1%)
Other Toxicity	0 (0.0%)	0 (0.0%)	9 (23.7%)	3 (7.1%)	12 (8.7%)
Dose delay/reduction within 1st 4 cycles of treatment^b^	14 (51.9%)	4 (12.9%)	17 (44.7%)	8 (19.0%)	43 (31.2%)

a Data reported as mean ± SD for patient characteristics.

b Rates of toxicities resulting in dose reduction or termination of treatment (DLT).

**Figure 2:**
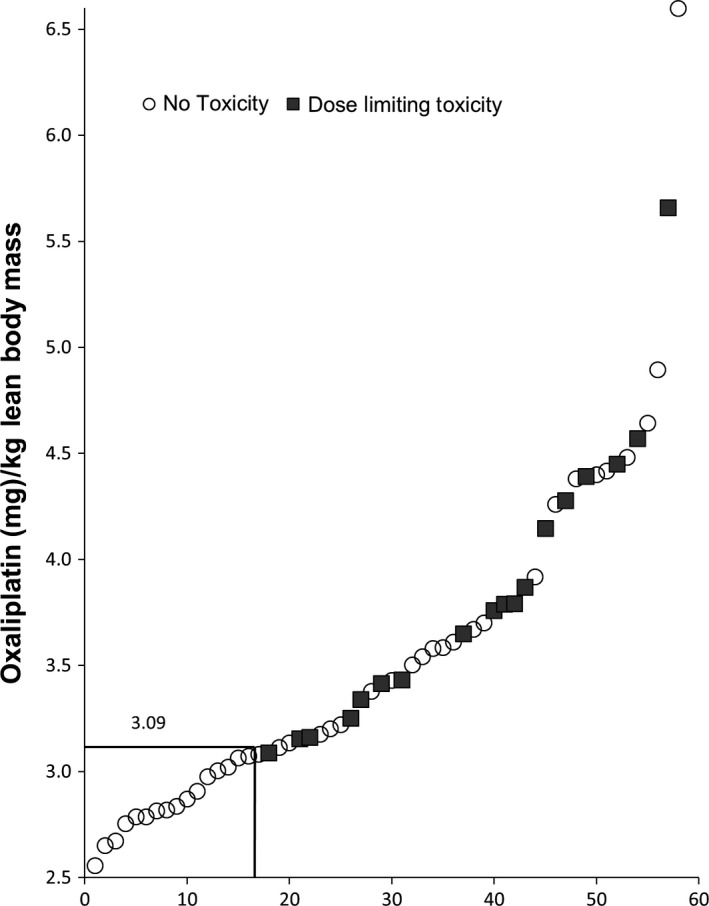
Distribution of the estimated oxaliplatin dose/kg lean body mass in French patients, from lowest to highest value. Estimated mg oxaliplatin/kg lean body mass for FRENCH population cohort (*n *=* *58) varied from 2.5 to more than 6.0 mg/kg. A value of 3.09 mg/kg LBM was determined to be the cut point for dose‐limiting toxicity (Area under ROC curve = 0.708). Toxicity rates were 0/17 (0.0%) and 18/41 (44.0%) using this cut point to separate the data into two groups (*P *=* *0.005; Fisher's Exact Test).

Patients had a very wide range of body composition and when the oxaliplatin dose was divided by estimated LBM value, the dose/LBM varied from 2.55 to 6.60 mg/kg LBM (Fig. 2). A cut point of 3.09 mg oxaliplatin/kg LBM was the threshold for developing DLT; 41 patients had oxaliplatin/kg LBM equal to or higher than this value. The mean value of oxaliplatin/kg LBM was different between the two groups below and above the cut point (3.88 versus 2.86 mg/kg LBM; +35.7%, *P *<* *0.001, Student's *t* test) (Table 2). This was true of all elements of the regimen, 5FU/kg LBM was also higher *P *<* *0.001) and for those patients who had irinotecan or cetuximab‐containing regimens both these drugs/kg LBM were higher (*P *<* *0.001) in patients who fell above the cut point. In contrast, the BSA differed by only 7% between the two groups (Table 2).

**Table 2:** Comparison of patients who received Oxaliplatin per kg LBM

Variables	FOLFOX Regimens France			FOLFOX Regimens Canada		
	<3.09 mg/kg LBM Oxaliplatin	≥3.09 mg/kg LBM Oxaliplatin	*P* value	<3.55 mg/kg LBMOxaliplatin	≥3.55 mg/kg LBM Oxaliplatin	*P* value
Number of patients	17	41	–	22	58	–
% Male	16 (94.1%)	15 (36.9%)	<0.001^b^	13 (81.8%)	24 (41.4%)	0.001^b^
Age	59.2 ± 7.8	60.1 ± 8.7	0.715	61.09 ± 11.5	63.4 ± 11.4	0.412
Disease Stage (I,II,III,IV) %	0, 0, 0, 100	0, 0, 0,100	–	7, 20, 53, 20	0, 4.0, 28, 68	–
Functional Status 0, 1, 2, 3 (%)	29, 71, 0, 0	34, 66, 0, 0	–	20, 73, 7, 0	28, 52, 12, 8	–
Weight (kg)	75.4 ± 12.3	66.5 ± 15.2	0.032	79.7 ± 15.6	73.4 ± 14.7	0.092
Height (m)	172 ± 8.46	164.8 ± 9.58	0.009	174.6 ± 8.0	167.2 ± 10.2	0.003
Body mass index (kg/m^2^)	25.3 ± 2.65	24.3 ± 4.24	0.370	26.1 ± 4.2	26.2 ± 4.7	0.871
Body surface area (cm^2^)	1.89 ± 0.19	1.74 ± 0.23	0.018	1.97 ± 0.21	1.82 ± 0.21	0.005
Total muscle cross‐sectional area at 3rd lumbar vertebra (cm^2^)	164.8 ± 22.2	119.5 ± 28.0	<0.001^a^	161.6 ± 28.6	123.1 ± 27.0	<0.001^a^
Whole‐body lean (kg)^c^	55.5 ± 6.7	41.9 ± 8.4	<0.001^a^	54.5 ± 8.6	43.0 ± 8.1	<0.001^a^
Oxaliplatin (mg/kg LBM)	2.86 ± 0.16	3.88 ± 0.73	<0.001^a^	3.21 ± 0.2	4.24 ± 0.4 0.5	<0.001^a^
5FU (mg/kg LBM)	94.3 ± 5.5	115.7 ± 17.2	<0.001^a^	97.6 ± 11.9	120.2 ± 13.7	<0.001^a^
Dose‐limiting toxicity during 1st four cycles, Number (%)	0 (0.0%)	18 (44.0%)	0.005^b^	3 (13.6%)	22 (37.9%)	0.024^b^
Early dose‐limiting neuropathy	0	9 (22%)	0.033^b^	0/22	9/58	0.046^b^

Data reported as mean ± SD for patient characteristics. LBM, lean body mass.

a Significant differences (Student's *T*‐Test).

b Significant differences (Fisher's Exact Test (*F*)).

c Calculated from regression equation Whole‐body lean tissue mass (kg) = [(L3 Muscle measured by CT (cm^2^) × 0.3) + 6.06].

The cut‐point analysis gave a clear separation of the risk of experiencing DLT which occurred in 18/41 (44.0%) patients above the cut point compared to 0/17 (0%) patients below this cut point (*P *<* *0.001; Fisher's Exact Test)(Fig. 2, Table 2). Patients above the cut point were more likely to be female but were not different in age than those below it. Lastly, none of the patients below the cut point developed neuropathy DLT within the first four cycles, whereas 9/41 (22%) of those above the cut point stopped or reduced treatment due to neurotoxicity within the first four cycles (*P *=* *0.033 Fisher's Exact Test).

### Canadian cohort

Table 1 describes patients' demographics, disease characteristics, chemotherapy regimens, body composition, and toxicities. For all FOLFOX regimens the administered dose was within ~5% of the target dose. Overall 31.2% of patients had treatment delays or dose reductions within the first four cycles, this occurred in 44.7% of women and 19.0% of men (*P *=* *0.009 Fisher's Exact Test).

The data for this sample were strikingly similar to the data from the French cohort, in terms of the overall body composition variation, the cut‐point value, discrimination of toxicity and the sex distribution above and below the cut point. Canadian patients also had a very wide range of body composition and when the oxaliplatin dose was divided by estimated LBM, the dose/LBM varied from 2.68 to 5.00 mg/kg LBM. A cut point of 3.55 mg oxaliplatin/kg LBM was the threshold for developing DLT determined by ROC analysis. Fifty‐eight patients had oxaliplatin/kg LBM ≥than this value (Table 2), and a DLT was experienced by 22 (37.9%) of these patients compared to 22 patients below this cut point in whom only 3 (13.6%) had DLT (*P *=* *0.024, Fisher's Exact Test). Lastly, none of the patients below the cut point developed peripheral neuropathy within the first four cycles, whereas in 9/58 (15.5%)of those above the cut point, had a neuropathy DLT (*P *=* *0.046).

### Combined French and Canadian data

**Figure 3:**
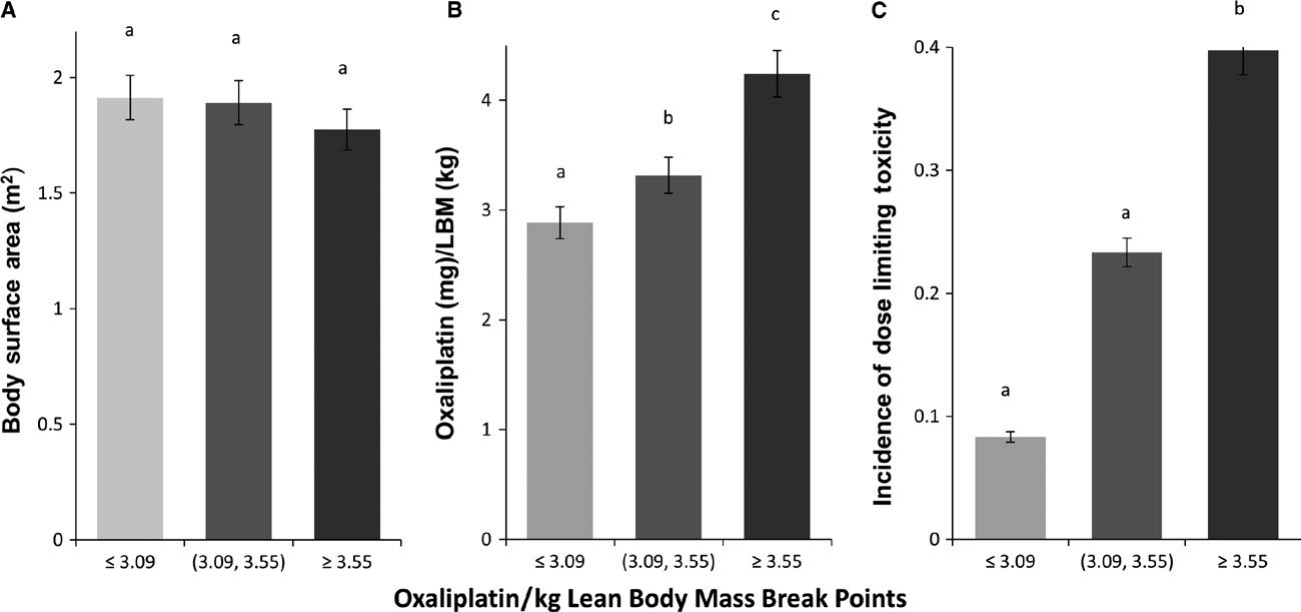
Combined characteristics of French and Canadian Populations. (*n *=* *138). Data were stratified into three groups based on the estimate of oxaliplatin dose/kg lean body mass: ≤3.09, between 3.09 and 3.55 and ≥3.55; ^a,b,c^ means with different superscripts are different, *P *<* *0.04.

Figure 3 represents features of the combined datasets (*n *=* *138). Data were stratified into three groups based on the estimate of oxaliplatin dose/kg LBM: ≤3.09, between 3.09 and 3.55 and ≥3.55. Notably, patients in these three groups were not different in BSA. The highest dose/LBM group had 39.9% DLT of which one quarter was neuropathy, whereas the lowest group had 8.3% DLT (*P *<* *0.01) of which none was neuropathy.

## Discussion

These results show that oxaliplatin doses normalized to LBM strongly discriminates an individual patient's likelihood of experiencing DLT, especially peripheral neuropathy. This was seen in two different populations, a sample of participants from a series of randomized clinical trials in France and a standard care population from Canada, yet giving findings that were strikingly similar. When drug dose/kg estimated LBM was subjected to a cut‐point analysis, essentially all DLTs occurred in patients who received greater than the cut‐point value. As well, all cases of dose‐limiting neuropathy occurred above the cut‐point value. There are two potential clinical implications of these findings—on the one hand there was a concentration of toxicity in patients above the cut point. Patients above the cut point were characterized by DLT in general, and there was a notable incidence of early neuropathy, possibly attributable to a higher cumulative exposure to oxaliplatin. Dose/kg LBM may add to our ability to assess risk of severe neuropathy which may be clinically unacceptable. On the other hand, there was negligible toxicity in the patients below the cut point. Oxaliplatin doses less than ~3.1 mg/kg LBM were associated with low risk of DLT, which raises questions as to whether patients so treated could have tolerated and benefitted from higher doses.

The studied patients in both populations had a wide distribution of body composition and specifically of LBM within any stratum of BSA. Considering both cohorts together variation in LBM renders up high variation in estimated oxaliplatin dose (range [2.55, 6.60] mg/kg LBM); the same was true for 5FU/kg LBM (range [66.2, 168.5] mg/kg LBM). This potential impact of LBM variation has not been fully appreciated. In the METHEP study, standard and high‐dose oxaliplatin treatments were studied, however, despite intentions to give two distinct drug levels (85 mg/m², or high dose 130 mg/m²),when body composition is taken into account the lines between these intended doses is blurred. For example of the 41 people in the French dataset whose oxaliplatin/kg LBM was above the cut point, 4 (16%) were on oxaliplatin 85 mg/m², not 130 mg/m² and their elevated dose (and toxicity) associated with their body composition (i.e., low LBM). Some people on oxaliplatin 85 mg/m² who had low LBM, got a higher drug dose/kg LBM than others on 130 mg/m² who had a high LBM relative to their BSA.

Several prior studies reported associations between CT‐derived assessments of LBM and chemotherapy toxicity 12, 13, 14, 15, 17, 24, 27, 28. These studies may be considered exploratory, and investigations were conducted mostly at single sites with relatively small numbers of patients. Skeletal muscle mass or LBM were related to the prevalence of DLT defined as treatment toxicity resulting in dose reduction or treatment discontinuation. The cancers included gastrointestinal, renal, lung, esophageal, thyroid, and breast. The cancer therapies studied included drugs dosed based on BSA including gemcitabine and vinorelbine 13, doxorubicin 27 capecitabine 17, and 5FU 12 as well as targeted therapies, which are normally flat dosed (all patients receive the same dose) including sunitinib, sorafenib, and vandetanib 14 as well as afatinib 15. One study included a group of patients who were participants in phase I clinical trials 28. Overall these studies were consistent in finding that reduced LBM had a significant association with increased incidence of DLT (in eight of nine studies in which this was evaluated). One early study concerned colorectal cancer 12 and this was in the context of 5FU monotherapy. Another study including a prospective analysis of 51 patients with metastatic colorectal cancer treated with a variety of chemotherapy, found that sarcopenia was the only significant factor associated with grade 3–4 toxicity on multivariate analysis 29. This study expands this theme in colorectal cancer to FOLFOX‐based regimens. It should be noted that at the time of treatment of patients in both cohorts, physicians, and patients were blind to the body composition status of the patients. One merit of this study is that the findings were validated in a second independent population. Our two populations were quite different. The French patients were from randomized studies with specific inclusion/exclusion criteria with standardized toxicity assessments and dose adjustment protocols. The Canadian patients received standard care). These differences could be a limitation but also a strength in the sense that both samples yielded very similar conclusions, in spite of small sample size.

Sex differences in body composition are well recognized, with women being notably less muscular than men and therefore having a lower LBM. As the majority of patients with low LBM relative to their BSA were female, we suggest that difference in or trends toward differential toxicity by sex may be partially explained by this feature of body composition. Previous studies have suggested that women experience more 5FU toxicity than men 30, 31, 32, 33 although they failed to provide a consistent explanation for this difference. Several mechanisms have been proposed, and one suggestion is that 5FU metabolism may be different in women 31. Dihydropyrimidine dehydrogenase (DPD) is believed to play a major role in 5FU catabolism. Some authors have suggested that women are prone to DPD deficiency 32, 33 but a larger study by Etienne et al. 34. reported that DPD deficiency is a rare event. 5FU clearance has also been suggested to be lower in women than in men 35, 36. An important proportion of the toxicity observed here in women was associated with a low LBM, and hence a higher dose of drug/LBM than men. Notably, the majority (87–96%) of patients who fell below toxicity cut points here were men.

There were some differences between the French and Canadian samples studied with respect to body weight and prevalence of sarcopenia, performance status, and disease stage distribution, but further work in larger populations is required to confirm if there are any regional differences. Cut‐point values were not identical in the two populations, however, this apparent difference should be treated with caution owing to the limiting sample size.

CT/MRI images provide the highest available precision and specificity in human body composition analysis 37. Amounts of adipose and lean tissues in single lumbar abdominal images correlate very well with whole‐body lean and adipose tissues 25. Image analysis is both a highly precise and a clinically expedient measure of body composition, and we argue that the CT‐defined metrics used here are essential because of the weak relationship between overall weight, BSA or BMI, and LBM. Using this approach, we have obtained data to suggest that differences in toxicities between patients may in part be due to variation in LBM. Previous research 3, 6 suggests that dose normalization to LBM may be a useful way to individualize chemotherapy. This concept awaits verification through prospective testing of drug dosing schedules per kg LBM.

## Conflict of Interest

**N**one declared.
